# *NECTIN4* Amplification Is a Frequent Event in Central Nervous System Metastases of Urothelial Carcinoma

**DOI:** 10.1016/j.euros.2026.02.009

**Published:** 2026-02-21

**Authors:** Richard Weiten, Constantin Rieger, Julian Heidenreich, Yuri Tolkach, Thomas Stehle, Florian Schmid, Teresa Schmidt, Martin Glas, Tobias Blau, Kathy Keyvani, Thomas Büttner, Viktor Grünwald, Michael Hölzel, Axel Heidenreich, Markus Eckstein, Niklas Klümper

**Affiliations:** a Department of Urology, Uro-Oncology, Robot-Assisted and Specialized Urologic Surgery, University Hospital Cologne, Cologne, Germany; b Institute of Pathology, University Hospital Cologne, Cologne, Germany; c Institute of Neuropathology, University Hospital Cologne, Cologne, Germany; d Department of Urology, University Hospital Zurich, Zurich, Switzerland; e Department of Neurology and Center for Translational Neuro- and Behavioral Sciences, University Hospital Essen, University Duisburg-Essen, Essen, Germany; f Department of Neurology and Neurooncology, St. Marien Hospital, Lünen, Germany; g Department of Neurooncology, Center for Neurology, University Hospital Bonn, Bonn, Germany; h Institute of Neuropathology, University Hospital Essen, Essen, Germany; i Department of Urology and Paediatric Urology, University Hospital Bonn, Bonn, Germany; j Clinic for Internal Medicine (Tumor Research) and Clinic for Urology, Interdisciplinary Genitourinary Oncology at the West-German Cancer Center, University Hospital Essen, Essen, Germany; k Institute of Experimental Oncology, University Hospital Bonn, Bonn, Germany; l Department of Urology, Medical University Vienna, Vienna, Austria; m Institute of Pathology, University Hospital Erlangen, Friedrich-Alexander-Universität Erlangen-Nürnberg, Erlangen, Germany

**Keywords:** Antibody-drug conjugate, Central nervous system, Metastases, NECTIN4, Urothelial carcinoma

## Abstract

*NECTIN4* amplification has emerged as a promising biomarker for predicting the response to anti-NECTIN4 therapies in metastatic urothelial carcinoma (mUC). The anti-NECTIN4 antibody-drug conjugate enfortumab vedotin (EV) combined with pembrolizumab (EV/P) is now the standard of care for mUC, with results demonstrating that it prolongs overall survival in the perioperative setting for patients with muscle-invasive bladder cancer (MIBC). However, data on its effectiveness in patients with active central nervous system (CNS) metastases (MET) are limited, as these patients were excluded from pivotal trials. Recent studies show that *NECTIN4* amplification is frequent in mUC (∼15–25%) and predicts the response to single-agent EV and to EV/P. Furthermore, *NECTIN4* amplification appears to be a stable genomic feature during metastasis progression. So far, no research has specifically examined *NECTIN4* amplification across different metastatic sites, with no systematic study in CNS MET, which is associated with poor outcomes. We compared a CNS MET cohort (*n* = 18) to two previous comprehensive mUC cohorts (1) non-CNS mUC (*n* = 128) and (2) EV-treated mUC (*n* = 108). *NECTIN4* gene amplification was found in 67% of CNS MET cases (12/18), which is significantly higher than the 26% in the EV-treated cohort at baseline (28/108). This corresponds to an absolute difference of 41% (95% confidence interval 16–59%; *p* = 0.002). Consistently, membranous NECTIN4 expression was higher in CNS MET (median H score 175, interquartile range [IQR] 88–260) than in non-CNS mUC (median H score 40, IQR 0–140; *p* < 0.001). These findings provide a strong biological rationale for extending the use of NECTIN4-targeted therapies such as EV to patients with brain MET.

**Patient summary:**

We found that tumor samples from patients with cancer of the urinary tract that had spread to the brain had higher expression of a gene called NECTIN4 (67%). Our findings suggest that treatments targeting NECTIN4, such as a drug called enfortumab vedotin with or without pembrolizumab, might benefit patients with brain metastases, especially if their tumors have high NECTIN4 levels.

Central nervous system (CNS) metastases (MET) in urothelial carcinoma (UC) remain a rare but increasingly recognized clinical challenge and are associated with poor prognosis and limited therapeutic options [1]. Historically, the incidence of CNS involvement was approximately 1%, but recent data indicate that rates have risen to 3–16%, which probably reflect improvement in systemic disease control and prolonged survival because of advances in chemotherapy, immune checkpoint inhibitors, and targeted therapies [2].

Standard management of CNS MET includes local therapies such as surgical resection and/or radiotherapy (either stereotactic radiosurgery or whole-brain radiotherapy), as systemic therapy has historically shown limited CNS activity, largely because of poor drug penetration across the blood-brain barrier (BBB) [3].

Recent advances in systemic therapy, particularly the development of antibody-drug conjugates (ADCs), are transforming the management of various cancers [4], including metastatic UC (mUC) [5], [6]. Notably, the TUXEDO-1 trial demonstrated robust activity of the anti-HER2 ADC trastuzumab deruxtecan (T-DXd) in patients with HER2-positive breast cancer (BC) and active brain MET. In this prospective phase2 trial, there was a high intracranial response rate (IRR) of 73% to T-DXd with durable disease control, which underscores the potential role of ADCs in the management of CNS MET [7], [8].

Enfortumab vedotin (EV), an ADC targeting NECTIN4, is approved for single-agent therapy in patients with pretreated mUC and, more recently, in the first-line setting in combination with pembrolizumab [5], [6]. Notably, Vulsteke et al [9] reported the first clinical evidence of EV efficacy against active CNS MET in mUC, with marked intracranial responses and durable disease control in a small case series. Despite these advances, pivotal trials of EV have systematically excluded patients with active CNS MET, so the efficacy and safety of NECTIN4-directed therapies in this population are largely unknown [5], [6].

One emerging predictive biomarker for EV response is *NECTIN4* gene amplification, which is observed in approximately 20–30% of mUC cases [10], [11], [12]. *NECTIN4* amplification is a stable genomic alteration during metastatic progression and correlates with elevated membranous NECTIN4 protein expression, which is essential for EV binding and cytotoxicity [10]. Importantly, the predictive utility of *NECTIN4* amplification extends beyond mUC; it has been validated as a predictive biomarker in triple-negative breast cancer and non–small-cell lung cancer, in which high NECTIN4 levels are associated with poor prognosis and greater sensitivity to NECTIN4-targeted therapies. In both malignancies, the investigational bicyclic peptide-drug conjugate zelenectide pevedotin (formerly known as BT8009) has shown enhanced antitumor activity in *NECTIN4*-amplified disease [13], [14]. These findings led to the initiation of two biomarker-driven phase 2 clinical trials (NCT06840483 and NCT06933329) conducted by Bicycle Therapeutics, both of which have received US Food and Drug Administration fast track designation, in efforts to further define the role of *NECTIN4* amplification as a pan-cancer predictive biomarker for NECTIN4-directed therapies.

While the expression patterns and copy number status of *NECTIN4* have been comprehensively characterized in primary UC and in non-CNS metastatic sites, data regarding the *NECTIN4* expression and genomic status in CNS MET remain scarce. To address this knowledge gap, we investigated NECTIN4 protein expression and gene amplification using the methodology previously described [10] in a group of 18 patients with mUC and CNS MET (Supplementary Table 1), and compared the results to previously reported findings in two mUC cohorts: (1) 128 patients with non-CNS mUC [15] and (2) 108 patients with EV-treated mUC [10].

Representative immunohistochemical images in Figure 1A show patterns of membranous NECTIN4 expression in CNS MET samples. Of note, 67% (*n* = 12/18) of the cases had *NECTIN4* amplification (NECTIN4/CEN1 ratio ≥2.0), which is significantly higher than the amplification frequency of 26% at baseline in the EV-treated mUC cohort (*n* = 28/108). This corresponds to an absolute difference of 41% (95% confidence interval [CI] 16–59%; χ^2^ test, *p* = 0.002) [10]. Membranous NECTIN4 expression was enhanced in *NECTIN4*-amplified tumors (median H score 215, interquartile range [IQR] 122–275; vs 90, IQR 14–170; Mann-Whitney U test, *p* = 0.026; Fig. 1B). Overall, 72% of CNS lesions exhibited moderate to strong membranous NECTIN4 staining (previously defined as a H score of 100–300) [10]. The median H score for membranous NECTIN4 expression was significantly higher in the CNS MET cohort (175, IQR 88–260) than in the non-CNS MET cohort (40, IQR 0–140; Mann-Whitney U test, *p* < 0.001; Fig. 1C). These findings suggest a higher prevalence of *NECTIN4* amplification in CNS MET in comparison to other metastatic sites, and are consistent with recent genomic studies suggesting that *NECTIN4* amplification is a recurrent and stable event in mUC that is frequently associated with elevated membranous NECTIN4 protein expression [10]. However, the relatively small sample size of our CNS MET cohort (*n* = 18) limits statistical precision and should be considered when interpreting these findings.

**Fig. 1:**
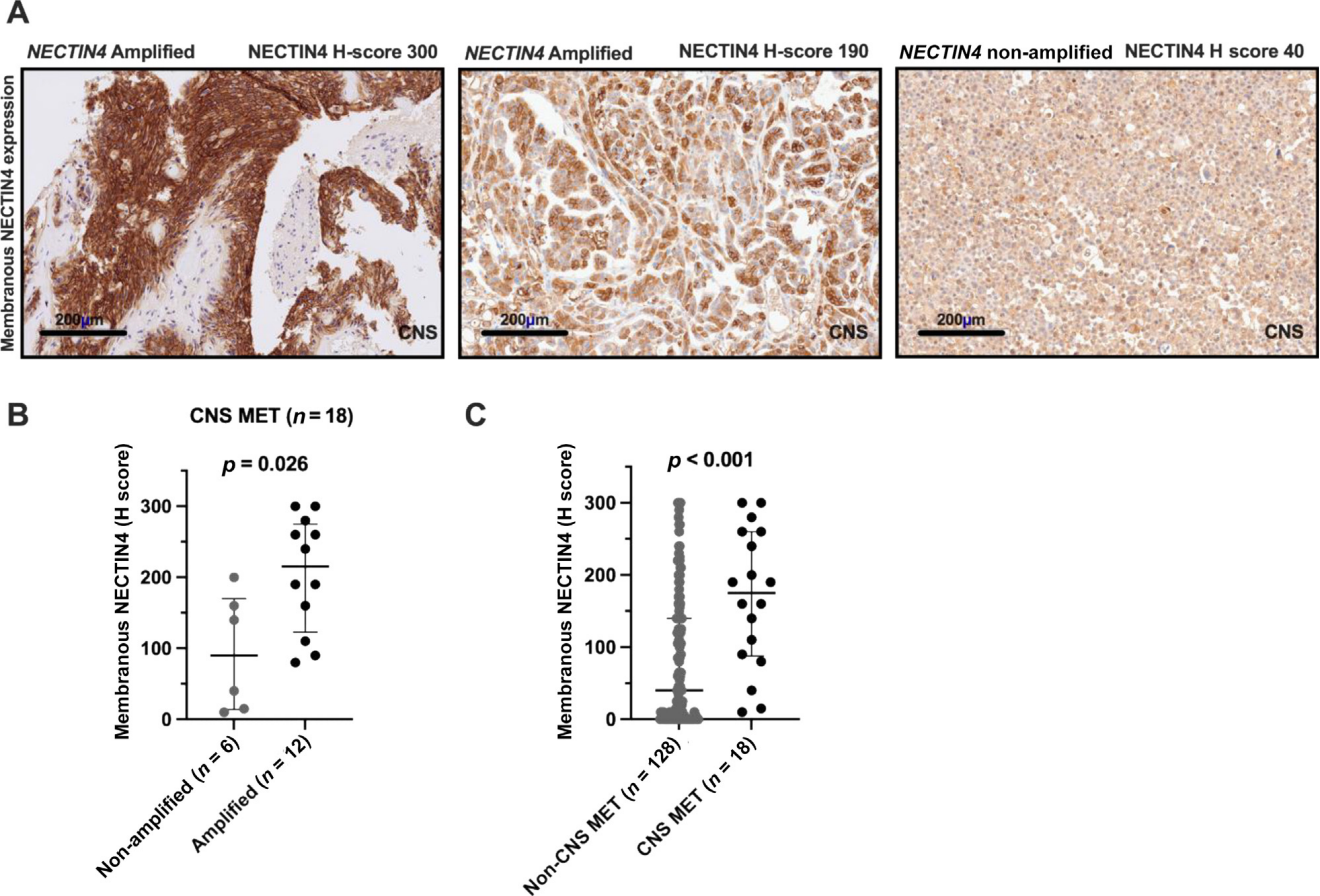
NECTIN4 expression and amplification in metastatic urothelial carcinoma with and without central nervous system (CNS) metastases (MET). (A) Representative immunohistochemistry images showing membranous *NECTIN4* staining and gene amplification in CNS MET. (B) NECTIN4 protein expression stratified by *NECTIN4* gene amplification status in the CNS MET group; amplified cases exhibited significantly higher H scores in comparison to non-amplified cases. (C) Quantitative comparison of NECTIN4 expression (H score) between CNS MET (*n* = 18) and non-CNS MET (*n* = 128) samples; the CNS MET group had significantly higher NECTIN4 expression.

These observations might be clinically relevant for several reasons. First, NECTIN4-targeted therapies, particularly EV, are now the standard of care in mUC after platinum-based therapy and immune checkpoint inhibition, and are approved in combination with pembrolizumab in the first-line setting [5], [6]. However, their use in patients with CNS involvement has been limited by exclusion criteria in registration trials (eg, EV-301 and EV-302). Our findings suggest that NECTIN4 remains highly expressed and frequently amplified in CNS MET, which provides a strong biological rationale for exploring the efficacy of EV in this population.

Second, recent translational studies have established *NECTIN4* amplification as a predictive biomarker for EV response [10]. Our group demonstrated that *NECTIN4*-amplified mUC cases were more likely to respond to both single-agent EV and EV/P, which was associated with longer progression-free and overall survival [10], [11]. Our observation that more than half of CNS MET cases harbor *NECTIN4* amplification suggests that patients with brain MET could be particularly responsive to NECTIN4-directed therapy such as EV. This is particularly relevant given the historically poor prognosis and limited treatment options for patients with UC and CNS involvement [1].

Third, the potential of ADCs for CNS MET treatment is an area of increasing clinical interest. While the intact BBB restricts the delivery of large molecules, metastatic lesions can disrupt the BBB, allowing macromolecular agents such as ADCs to reach therapeutic concentrations within the CNS compartment [7], [16]. This disruption is supported by both preclinical and clinical data, which have demonstrated that ADCs can achieve intracranial activity when the BBB is compromised [7], [16]. Recent clinical evidence highlights the efficacy of ADCs in this context: T-DXd has demonstrated high IRR (>70%) in patients with HER2-positive BC and active brain MET [7], [8], and Vulsteke et al [9] reported the first evidence of EV activity in patients with mUC and CNS involvement. However, prospective studies are needed to confirm *NECTIN4* amplification as a promising genomic biomarker for predicting clinical benefit from EV. To this end, we have initiated the observational EVOKE trial (DRKS-ID: DRKS00034745) for prospective validation.

In conclusion, *NECTIN4* amplification is highly prevalent in mUC with CNS MET. Our results provide a strong biological rationale for extending the use of NECTIN4-targeted therapies such as EV to patients with brain MET. Given the predictive relevance of *NECTIN4* amplification and the urgent therapeutic need in this population, prospective trials evaluating the CNS activity of EV (with or without pembrolizumab) and related agents are warranted.

  ***Author contributions:*** Richard Weiten had full access to all the data in the study and takes responsibility for the integrity of the data and the accuracy of the data analysis.

  *Study concept and design*: Weiten, Eckstein, Klümper.

*Acquisition of data*: Weiten, Rieger, J. Heidenreich, Stehle, Schmidt, Glas, Blau, Keyvani.

*Analysis and interpretation of data*: Weiten, Tolkach, Stehle, Eckstein, Klümper.

*Drafting of the manuscript*: Weiten, Eckstein, Klümper.

*Critical revision of the manuscript for important intellectual content*: Rieger, Heidenreich, Tolkach, Stehle, Schmid, Schmidt, Glas, Blau, Keyvani, Büttner, Grünwald, Hölzel, Heidenreich.

*Statistical analysis*: Weiten, Eckstein, Klümper.

*Obtaining funding*: None.

*Administrative, technical, or material support*: Tolkach, Stehle, Eckstein.

*Supervision*: A. Heidenreich, Eckstein, Klümper.

*Other*: None.

  ***Financial disclosures:*** Richard Weiten certifies that all conflicts of interest, including specific financial interests and relationships and affiliations relevant to the subject matter or materials discussed in the manuscript (eg, employment/affiliation, grants or funding, consultancies, honoraria, stock ownership or options, expert testimony, royalties, or patents filed, received, or pending), are the following: Constantin Rieger reports honoraria from Medac and Astellas. Thomas Büttner reports personal fees/speaker honoraria from Astellas; a consulting or advisory role for Merck; and travel and accommodation expenses from MSD and Ipsen. Viktor Grünwald reports research funding from AstraZeneca, Novartis, BMS, MSD, Ipsen, and Pfizer; honoraria and consultation fees from AstraZeneca, BMS, Novartis, Amgen, Astellas, Apogepha, Ipsen, EISAI, MSD, MerckSerono, Roche, EUSA Pharm, Janssen, ONO Pharmaceutical, Cureteq, Debiopharm, PCI Biotech, Oncorena, Novartis/AAA, and Gilead; stocks in AstraZeneca, BMS, SeaGen, MSD, and GenMab; and travel expenses from AstraZeneca, BMS, MerckSerono, and Janssen. Michael Hölzel reports research funding from TME Pharma (Noxxon); and honoraria from BMS and Novartis. Markus Eckstein reports honoraria from AstraZeneca, Roche, Astellas, Genomic Health, Janssen, Owkin, Diaceutics, Ferring, Eisai, Merck, Bicycle Therapeutics, Zytomed Systems, and Lilly; consulting or advisory roles for AstraZeneca, Janssen, Genomic Health, Diaceutics, Gilead Sciences, Owkin, MSD Oncology, Merck, Bicycle Therapeutics, Lilly, and Ferring; speaker bureau participation for Diaceutics, Roche, AstraZeneca, MSD, Astellas, Merck, Bicycle Therapeutics, MSD, Zytomed Systems, Eisai, and BMS; research funding from STRATIFYER, Janssen/Johnson & Johnson, AstraZeneca/MedImmune, Owkin, Gilead Sciences, Bicycle Therapeutics, QuiP GmbH, and Cepheid; travel and accommodation expenses from AstraZeneca, Roche, MSD, Janssen-Cilag, Genomic Health, Diaceutics, Astellas Pharma, Ferring, Eisai, Merck, Bicycle Therapeutics, Cepheid, and BMS; stock options in Bicycle Therapeutics; and an interest in patent PCT/US2024/051200. Niklas Klümper reports stock and other ownership interests in Bicycle Therapeutics; personal fees/speaker honoraria from Astellas, Novartis, Ipsen, Photocure, MSD, and Merck; consulting or advisory roles for Astellas, Eisai, Merck, MSD, and Bicycle Therapeutics; research funding from Bicycle Therapeutics; and travel and accommodation expenses from Astellas, Novartis, Ipsen, Photocure, MSD, Merck, and Johnson & Johnson. All other authors declare no conflict of interest.

  ***Funding/Support and role of the sponsor:*** No specific funding was obtained for this study.

  ***Acknowledgments:*** Richard Weiten is supported by Ferdinand Eisenberger grant WeR1/FE-22 from Deutsche Gesellschaft für Urologie. Michael Hölzel is funded by Deutsche Forschungsgemeinschaft under Germany’s Excellence Strategy (EXC2151–390873048) and is a member of the CANTAR project, which receives funding from the Netzwerke 2021 program, an initiative of the Ministry of Culture and Science of the State of North Rhine-Westphalia. Markus Eckstein is supported by the Else Kröner-Fresenius Foundation (2020_EKEA.129; 2023_EKES.07), an Else Kröner-Fresenius Clinician Scientist Professorship (2024_EKCS.08), German Cancer Aid/Deutsche Krebshilfe (70116726, 70116777/70116778), the US Department of Defense (CA230411), Wilhlem-Sander-Stiftung (Nr. 2024.022.1), the clinician scientist program of Interdisziplinäres Zentrum für Klinische Forschung of Friedrich-Alexander Universität (IZKF-FAU), the TOPeCS T04 funding line from IZKF-FAU, advanced research grant D41 from IZKF-FAU Erlangen-Nürnberg, and a Young Clinical Scientist Fellowship from the Bavarian Center for Cancer Research (BZKF YSF-TP01). Open-access funding is enabled and organized by the DEAL project. Niklas Klümper is supported by the Advanced Clinician Scientist Program Bonn of the Medical Faculty of the University of Bonn (grant 01EO2107).

  ***Ethics statement:*** This study was approved by the ethics committee of the Medical Faculty of the University of Cologne (approval number 23-1178) and conducted in accordance with the Declaration of Helsinki. All patients provided written informed consent.

  ***Data sharing statement:*** The data that support the study findings are available from the corresponding author on reasonable request.
