# Supplementary material for: NECTIN4 Amplification Is a Frequent Event in Central Nervous System Metastases of Urothelial Carcinoma
Source: Eur Urol Open Sci. 2026 Feb 21;86:42–6. doi: 10.1016/j.euros.2026.02.009 (PMC12936467; doi:10.1016/j.euros.2026.02.009)
Supplement: Supplementary Data 1 [file mmc1.docx]

**Supplementary material**

**Multicenter study cohort**

This was a retrospective analysis of a multicenter cohort comprising patients with metastatic urothelial carcinoma (mUC) treated at university hospitals in Cologne, Essen, and Erlangen. The patients had central nervous system (CNS) metastases (MET; *n* = 18) or non-CNS metastatic disease (*n* = 128). Clinicopathological characteristics for all patients are detailed in Supplementary Table 1.

Histopathological classification was performed according to the 8th edition of the TNM classification of malignant tumors and the 5th edition of the World Health Organization classification for urogenital tumors, reflecting current standards for mUC diagnosis and staging.

The study protocol was approved by the ethics committees of Friedrich-Alexander-University Erlangen-Nürnberg (approval numbers 329_16B and 97_18Bc), University Hospital Essen (approval number 23-11477-BO), and the medical faculty at the University of Cologne (approval number 23-1178). The study was conducted in accordance with the Declaration of Helsinki. Written informed consent was obtained from all participants before study inclusion.

**NECTIN4 immunohistochemistry**

Immunohistochemical analysis of NECTIN4 was performed using a BOND-MAX autostainer (Leica Biosystems, Wetzlar, Germany) following established protocols. A monoclonal anti-NECTIN4 antibody (catalog number ab251110, Abcam, Cambridge, UK) was applied at a dilution of 1:800, with incubation for 32 min at 37°C, as previously described [1].

Membranous NECTIN4 expression was independently evaluated by two board-certified pathologists (Y.T. and M.E.) using the H-score method, which integrates staining intensity and the percentage of positive tumor cells. Staining was categorized as negative (H score 0–14), weak (H score 15–99), moderate (H score 100–199), or strong (H score 200–300), in accordance with established criteria for NECTIN4 assessment in UC [1].

***NECTIN4* fluorescence in situ hybridization**

The *NECTIN4* gene copy number was assessed via fluorescence in situ hybridization using a commercially available probe (NECTIN4-20-GR; Empire Genomics, Buffalo, NY, USA) that specifically targets the *NECTIN4* locus (NCBI gene ID 81607) on chromosome 1q23.3. The probe consists of a fluorescently labeled DNA sequence designed for high specificity to the *NECTIN4* gene, as previously described [2].

All hybridizations were performed in an accredited clinical molecular pathology laboratory (DIN EN ISO/IEC 17020 accreditation) according to standardized protocols to ensure analytic validity and reproducibility. Slides were evaluated using a fluorescence microscope equipped with appropriate filter sets for the detection of *NECTIN4* and centrin 1 (*CEN1*) signals. Representative tumor regions for formal analysis were selected by an experienced board-certified pathologist (M.E.) who was blinded to clinical outcomes to minimize selection bias and ensure objective assessment. The *NECTIN4*/*CEN1* ratio was calculated for each case, with tumors classified as having *NECTIN4* amplification if the ratio was ≥2.0. Tumors with a ratio <2.0 were considered to have no *NECTIN4* amplification, as previously described [2].

**Statistical analysis**

Statistical analyses were conducted using GraphPad Prism v9.4.0. To address the predefined study questions, the nonparametric Mann-Whitney U test was used to compare *NECTIN4* expression between tumors with and without amplification, and between the non-CNS and CNS MET groups. The difference in *NECTIN4* amplification frequency between the non-CNS and CNS MET groups was assessed using the χ^2^ test. All *p* values were calculated as two-sided, and statistical significance was defined as *p* < 0.05.

**References**

[1] Klümper N, Ralser DJ, Ellinger J, et al. Membranous NECTIN-4 expression frequently decreases during metastatic spread of urothelial carcinoma and is associated with enfortumab vedotin resistance. Clin Cancer Res 2023;29:1496–505. https://doi.org/10.1158/1078-0432.Ccr-22-1764

[2] Klümper N, Zschäbitz S, Büttner T, et al. 3087P NECTIN4 amplification as a predictive biomarker of response to enfortumab vedotin plus pembrolizumab in first-line metastatic urothelial carcinoma: a multicenter cohort study. Ann Oncol 2025;36:S1600. https://doi.org/10.1016/j.annonc.2025.08.3701

[3] Erlmeier F, Klümper N, Landgraf L, et al. Spatial immunephenotypes of distant metastases but not matched primary urothelial carcinomas predict response to immune checkpoint inhibition. Eur Urol 2023;83:133–42. https://doi.org/10.1016/j.eururo.2022.10.020

**Supplementary Table 1 – Clinicopathological characteristics of the study cohort**

| Parameter | Non-CNS MET  (*n* = 128) ^a^ | CNS MET  (*n* = 18) | *p* value ^b^ |
| --- | --- | --- | --- |
| Median age at diagnosis, yr (interquartile range) | 71 (63–76) | 64 (60, 68) | <0.001 |
| Male sex, *n* (%) | 95 (74) | 14 (78) | >0.9 |
| Primary tumor, *n* (%) |  |  | <0.001 |
| Urothelial bladder cancer | 126 (98) | 13 (72) |  |
| Upper tract urothelial carcinoma | 2 | 5 |  |
| Previous systemic therapies, *n* (%) ^c^ |  |  | 0.8 |
| ≤1 | 80 (63) | 12 (67) |  |
| ≥2 | 48 | 6 |  |
| CNS = central nervous system; MET = metastases.  ^a^ This cohort was previously described by Erlmeier et al [3]  ^b^ Wilcoxon rank-sum test for continuous variables; Fisher’s exact test for categorical variables.  ^c^ Chemotherapy or an immune checkpoint inhibitor. | | | |
